# Lassa virus isolates from Mali and the Ivory Coast represent an emerging fifth lineage

**DOI:** 10.3389/fmicb.2015.01037

**Published:** 2015-10-01

**Authors:** John T. Manning, Naomi Forrester, Slobodan Paessler

**Affiliations:** Department of Pathology, University of Texas Medical BranchGalveston, TX, USA

**Keywords:** Lassa virus, Lassa fever, lineage, genetic diversity, phylogenetics

## Abstract

Previous imported cases of Lassa fever (LF) into the United Kingdom from the Ivory Coast and Mali, as well as the detection of Lassa virus (LASV) among the *Mastomys natalensis* population within Mali has led to the suggestion that the endemic area for LF is expanding. Initial phylogenetic analyses arrange isolates from Mali and the Ivory Coast separately from the classical lineage IV isolates taken from Sierra Leone, Guinea, and Liberia. The availability of full genome sequences continues to increase, allowing for a more complete phylogenetic comparison of the isolates from Mali and the Ivory Coast to the other existing isolates. In this study, we utilized a Bayesian approach to infer the demographic histories of each LASV isolate for which the full sequence was available. Our results indicate that the isolates from Mali and the Ivory Coast group separately from the isolates of lineage IV, comprising a distinct fifth lineage. The split between lineages IV and V is estimated to have occurred around 200–300 years ago, which coincides with the colonial period of West Africa.

## Introduction

Lassa virus (LASV) is the causative agent of Lassa fever (LF), a potentially fatal disease that infects as many as 100,000 people annually in endemic areas. Since the discovery of the virus in 1969, the endemic area for LASV has been mapped to the West African countries of Nigeria, Sierra Leone, Guinea, and Liberia (Ogbu et al., 2007). The primary natural host, *Mastomys natalensis*, is distributed throughout West Africa despite the constricted endemic area of LF, and infected rodents are distributed focally within the endemic area (Demby et al., 2001; Lecompte et al., 2006). However, recent cases of LF within the West African countries of Mali and the Ivory Coast suggest that the endemic area is expanding (Atkin et al., 2009; Sogoba et al., 2012).

The virus genome consists of an L and S segment, which encode the RNA-dependent RNA polymerase (LP), matrix (Z) protein, nucleoprotein (NP), and the glycoprotein precursor (GPC). Phylogenetic analyses of either partial or full-length LASV protein sequences have revealed that four lineages exist among LASV isolates. The four lineages correlate strongly with the geographic point of origin for the respective isolates (Bowen et al., 2000). While each lineage can be distinctly delineated from one another in phylogenetic analyses of each full-length gene (with the exception of the small Z protein), there is some variability in the relationships between individual lineages for each gene (Ehichioya et al., 2011). With full-length LASV sequences becoming more readily available, more extensive phylogenetic analyses utilizing full-length genes over a longer time frame can be performed, allowing for more complete characterization of each individual lineage.

LASV has been suggested to have arrived in the Sierra Leone region from Nigeria between 150 and 250 years ago due to movement within the colonial period, and the recent emergence of LASV within Mali and the Ivory Coast has been suggested to be caused by movement during the Sierra Leone civil war between 1991 and 2002 (Lalis et al., 2012). The prototypic strain from the Ivory Coast, AV, was reported in 2000 as a case imported into Germany (Gunther et al., 2000). Although cases of LF have been reported in the Ivory Coast and Mali, sequencing data for isolates from these countries has only become available within the last few years (Safronetz et al., 2010, 2013). While the sequence data for these isolates has become readily available, the genetic relationship of these isolates to the classical lineages has not been completely characterized. The purpose of this study was to determine whether a fifth lineage is emerging within Mali and the Ivory Coast using analyses of complete LP, NP, and GPC genes. Using Bayesian analysis, we investigated the relationship of isolates from Mali and the Ivory Coast to all available isolates within the four traditional lineages.

## Methods

### Sequence alignments

Full-length L and S segments for each available isolate were imported into SeaView 4 (Gouy et al., 2010) from GenBank (Table 1), and nucleotide sequences were aligned as amino acids using MUSCLE (Edgar, 2004), and subsequently converted back to nucleotide sequences in order to maintain third-nucleotide alignment. The resulting alignments were trimmed to include only ORFs for LP, NP, and GPC sequences and exported as nexus files for phylogenetic analysis.

**Table 1:** **Demographic information for each LASV isolate used in the analysis**.

**Isolate**	**Accession number (L segment)**	**Accession number (S segment)**	**Country of origin**	**Host**	**Year of isolation**
LASV1016-NIG-2009	KM822108.1	KM822109.1	Nigeria	Human	2009
LASV1015-NIG-2009	KM822106.1	KM822107.1	Nigeria	Human	2009
LASV1011-NIG-2009	KM822104.1	KM822105.1	Nigeria	Human	2009
LASV1008-NIG-2009	KM822102.1	KM822103.1	Nigeria	Human	2009
LASV1000-NIG-2009	KM822100.1	KM822101.1	Nigeria	Human	2009
LASV993-NIG-2009	KM822098.1	KM822099.1	Nigeria	Human	2009
LASV992-NIG-2009	KM822096.1	KM822097.1	Nigeria	Human	2009
LASV991-NIG-2009	KM822092.1	KM822095.1	Nigeria	Human	2009
LASV989-NIG-2009	KM822090.1	KM822091.1	Nigeria	Human	2009
LASV982-NIG-2009	KM822087.1	KM822088.1	Nigeria	Human	2009
LASV981-NIG-2009	KM822085.1	KM822086.1	Nigeria	Human	2009
LASV979-NIG-2009	KM822083.1	KM822084.1	Nigeria	Human	2009
LASV978-NIG-2009	KM822081.1	KM822082.1	Nigeria	Human	2009
LASV977-NIG-2009	KM822079.1	KM822080.1	Nigeria	Human	2009
LASV976-NIG-2009	KM822077.1	KM822078.1	Nigeria	Human	2009
LASV975-NIG-2009	KM822075.1	KM822076.1	Nigeria	Human	2009
LASV971-NIG-2009	KM822073.1	KM822074.1	Nigeria	Human	2009
LASV967-NIG-2009	KM822070.1	KM822071.1	Nigeria	Human	2009
LASV966-NIG-2009	KM822068.1	KM822069.1	Nigeria	Human	2009
LASV737-NIG-2009	KM822064.1	KM822065.1	Nigeria	Human	2009
LASV719-NIG-2009	KM822061.1	KM822062.1	Nigeria	Human	2009
LASV711-NIG-2009	KM822058.1	KM822059.1	Nigeria	Human	2009
LASV274-NIG-2010	KM822056.1	KM822057.1	Nigeria	Human	2010
LASV271-NIG-2010	KM822054.1	KM822055.1	Nigeria	Human	2010
LASV267-NIG-2010	KM822052.1	KM822053.1	Nigeria	Human	2010
LASV263-NIG-2011	KM822050.1	KM822051.1	Nigeria	Human	2011
LASV256-NIG-2010	KM822048.1	KM822049.1	Nigeria	Human	2010
LASV254-NIG-2011	KM822046.1	KM822047.1	Nigeria	Human	2011
LASV253-NIG-2011	KM822044.1	KM822045.1	Nigeria	Human	2011
LASV251-NIG-2010	KM822042.1	KM822043.1	Nigeria	Human	2010
LASV250-NIG-2011	KM822040.1	KM822041.1	Nigeria	Human	2011
LASV246-NIG-2010	KM822038.1	KM822039.1	Nigeria	Human	2010
LASV245-NIG-2011	KM822036.1	KM822037.1	Nigeria	Human	2011
LASV242-NIG-2010	KM822034.1	KM822035.1	Nigeria	Human	2010
LASV239-NIG-2010	KM822030.1	KM822031.1	Nigeria	Human	2010
LASV237-NIG-2010	KM822028.1	KM822029.1	Nigeria	Human	2010
LASV229-NIG-2010	KM822026.1	KM822027.1	Nigeria	Human	2010
LASV225-NIG-2010	KM822024.1	KM822025.1	Nigeria	Human	2010
LASV221-NIG-2010	KM822021.1	KM822022.1	Nigeria	Human	2010
LASV063-NIG-2009	KM822019.1	KM822020.1	Nigeria	Human	2009
LASV058-NIG-2008	KM822017.1	KM822018.1	Nigeria	Human	2008
LASV056-NIG-2008	KM822015.1	KM822016.1	Nigeria	Human	2008
LASV052-NIG-2008	KM822013.1	KM822014.1	Nigeria	Human	2008
LASV049-NIG-2009	KM822011.1	KM822012.1	Nigeria	Human	2009
LASV046-NIG-2009	KM822009.1	KM822010.1	Nigeria	Human	2009
LASV045-NIG-2009	KM822007.1	KM822008.1	Nigeria	Human	2009
LASV042-NIG-2009	KM822005.1	KM822006.1	Nigeria	Human	2009
LASV006-NIG-2008	KM822001.1	KM822002.1	Nigeria	Human	2008
LASV003-NIG-2008	KM821999.1	KM822000.1	Nigeria	Human	2008
ISTH2376-NIG-2012	KM821997.1	KM821998.1	Irrua, Nigeria	Human	2012
ISTH2358-NIG-2012	KM821995.1	KM821996.1	Irrua, Nigeria	Human	2012
ISTH2334-NIG-2012	KM821993.1	KM821994.1	Irrua, Nigeria	Human	2012
ISTH2316-NIG-2012	KM821991.1	KM821992.1	Irrua, Nigeria	Human	2012
ISTH2312-NIG-2012	KM821989.1	KM821990.1	Irrua, Nigeria	Human	2012
ISTH2304-NIG-2012	KM821987.1	KM821988.1	Irrua, Nigeria	Human	2012
ISTH2271-NIG-2012	KM821985.1	KM821986.1	Irrua, Nigeria	Human	2012
ISTH2217-NIG-2012	KM821983.1	KM821984.1	Irrua, Nigeria	Human	2012
ISTH2129-NIG-2012	KM821981.1	KM821982.1	Irrua, Nigeria	Human	2012
ISTH2094-NIG-2012	KM821978.1	KM821979.1	Irrua, Nigeria	Human	2012
ISTH2066-NIG-2012	KM821975.1	KM821976.1	Irrua, Nigeria	Human	2012
ISTH2065-NIG-2012	KM821973.1	KM821974.1	Irrua, Nigeria	Human	2012
ISTH2061-NIG-2012	KM821970.1	KM821971.1	Irrua, Nigeria	Human	2012
ISTH2046-NIG-2012	KM821964.1	KM821965.1	Irrua, Nigeria	Human	2012
ISTH2042-NIG-2012	KM821962.1	KM821963.1	Irrua, Nigeria	Human	2012
ISTH2037-NIG-2012	KM821960.1	KM821961.1	Irrua, Nigeria	Human	2012
ISTH2031-NIG-2012	KM821958.1	KM821959.1	Irrua, Nigeria	Human	2012
ISTH2025-NIG-2012	KM821956.1	KM821957.1	Irrua, Nigeria	Human	2012
ISTH2020-NIG-2012	KM821954.1	KM821955.1	Irrua, Nigeria	Human	2012
ISTH2016-NIG-2012	KM821952.1	KM821953.1	Irrua, Nigeria	Human	2012
ISTH2010-NIG-2012	KM821950.1	KM821951.1	Irrua, Nigeria	Human	2012
ISTH1137-NIG-2011	KM821948.1	KM821949.1	Irrua, Nigeria	Human	2011
ISTH1129-NIG-2012	KM821946.1	KM821947.1	Irrua, Nigeria	Human	2012
ISTH1121-NIG-2012	KM821944.1	KM821945.1	Irrua, Nigeria	Human	2012
ISTH1111-NIG-2011	KM821942.1	KM821943.1	Irrua, Nigeria	Human	2011
ISTH1107-NIG-2012	KM821940.1	KM821941.1	Irrua, Nigeria	Human	2012
ISTH1096-NIG-2012	KM821938.1	KM821939.1	Irrua, Nigeria	Human	2012
ISTH1069-NIG-2011	KM821936.1	KM821937.1	Irrua, Nigeria	Human	2011
ISTH1064-NIG-2011	KM821934.1	KM821935.1	Irrua, Nigeria	Human	2011
ISTH1058-NIG-2011	KM821932.1	KM821933.1	Irrua, Nigeria	Human	2011
ISTH1048-NIG-2011	KM821930.1	KM821931.1	Irrua, Nigeria	Human	2011
ISTH1038-NIG-2011	KM821928.1	KM821929.1	Irrua, Nigeria	Human	2011
ISTH1003-NIG-2011	KM821926.1	KM821927.1	Irrua, Nigeria	Human	2011
ISTH0595-NIG-2011	KM821923.1	KM821924.1	Irrua, Nigeria	Human	2011
ISTH0531-NIG-2011	KM821921.1	KM821922.1	Irrua, Nigeria	Human	2011
ISTH0230-NIG-2011	KM821919.1	KM821920.1	Irrua, Nigeria	Human	2011
ISTH0073-NIG-2011	KM821917.1	KM821918.1	Irrua, Nigeria	Human	2011
ISTH0047-NIG-2011	KM821915.1	KM821916.1	Irrua, Nigeria	Human	2011
ISTH0012-NIG-2011	KM821913.1	KM821914.1	Irrua, Nigeria	Human	2011
ISTH0009-NIG-2011	KM821911.1	KM821912.1	Irrua, Nigeria	Human	2011
Z0948-SLE-2011	KM822131.1	KM822132.1	Sierra Leone	*M. natalensis*	2011
Z0947-SLE-2011	KM822129.1	KM822130.1	Sierra Leone	*M. natalensis*	2011
LM778-SLE-2012	KM822123.1	KM822124.1	Sierra Leone	*M. natalensis*	2012
LM776-SLE-2012	KM822121.1	KM822122.1	Sierra Leone	*M. natalensis*	2012
LM765-SLE-2012	KM822116.1	KM822117.1	Sierra Leone	*M. natalensis*	2012
LM395-SLE-2009	KM822114.1	KM822115.1	Sierra Leone	*M. natalensis*	2009
LM222-SLE-2010	KM822112.1	KM822113.1	Sierra Leone	*M. natalensis*	2010
LM032-SLE-2010	KM822110.1	KM822111.1	Sierra Leone	*M. natalensis*	2010
G3278-SLE-2013	KM821908.1	KM821909.1	Sierra Leone	Human	2013
G3248-SLE-2013	KM821905.1	KM821906.1	Sierra Leone	Human	2013
G3234-SLE-2013	KM821903.1	KM821904.1	Sierra Leone	Human	2013
G3229-SLE-2013	KM821901.1	KM821902.1	Sierra Leone	Human	2013
G3206-SLE-2013	KM821898.1	KM821899.1	Sierra Leone	Human	2013
G3170-SLE-2013	KM821896.1	KM821897.1	Sierra Leone	Human	2013
G3151-SLE-2013	KM821893.1	KM821894.1	Sierra Leone	Human	2013
G3148-SLE-2013	KM821891.1	KM821892.1	Sierra Leone	Human	2013
G3106-SLE-2013	KM821888.1	KM821889.1	Sierra Leone	Human	2013
G3010-SLE-2013	KM821881.1	KM821882.1	Sierra Leone	Human	2013
G2944-SLE-2012	KM821879.1	KM821880.1	Sierra Leone	Human	2012
G2868-SLE-2012	KM821873.1	KM821874.1	Sierra Leone	Human	2012
G2723-SLE-2012	KM821870.1	KM821871.1	Sierra Leone	Human	2012
G2612-SLE-2012	KM821866.1	KM821867.1	Sierra Leone	Human	2012
G2587-SLE-2012	KM821864.1	KM821865.1	Sierra Leone	Human	2012
G2565-SLE-2012	KM821862.1	KM821863.1	Sierra Leone	Human	2012
G2557-SLE-2012	KM821860.1	KM821861.1	Sierra Leone	Human	2012
G2554-SLE-2012	KM821858.1	KM821859.1	Sierra Leone	Human	2012
G2431-SLE-2012	KM821855.1	KM821856.1	Sierra Leone	Human	2012
G2427-SLE-2012	KM821853.1	KM821854.1	Sierra Leone	Human	2012
G2405-SLE-2012	KM821851.1	KM821852.1	Sierra Leone	Human	2012
G2392-SLE-2012	KM821849.1	KM821850.1	Sierra Leone	Human	2012
G2363-SLE-2012	KM821846.1	KM821847.1	Sierra Leone	Human	2012
G2295-SLE-2012	KM821840.1	KM821841.1	Sierra Leone	Human	2012
G2280-SLE-2012	KM821838.1	KM821839.1	Sierra Leone	Human	2012
G2263-SLE-2012	KM821836.1	KM821837.1	Sierra Leone	Human	2012
G2259-SLE-2012	KM821834.1	KM821835.1	Sierra Leone	Human	2012
G2222-SLE-2011	KM821831.1	KM821832.1	Sierra Leone	Human	2011
G2197-SLE-2011	KM821829.1	KM821830.1	Sierra Leone	Human	2011
G2184-SLE-2011	KM821827.1	KM821828.1	Sierra Leone	Human	2011
G2165-SLE-2011	KM821825.1	KM821826.1	Sierra Leone	Human	2011
G2147-SLE-2011	KM821823.1	KM821824.1	Sierra Leone	Human	2011
G2141-SLE-2011	KM821821.1	KM821822.1	Sierra Leone	Human	2011
G1960-SLE-2011	KM821819.1	KM821820.1	Sierra Leone	Human	2011
G1792-SLE-2011	KM821813.1	KM821814.1	Sierra Leone	Human	2011
G1774-SLE-2011	KM821811.1	KM821812.1	Sierra Leone	Human	2011
G1727-SLE-2011	KM821809.1	KM821810.1	Sierra Leone	Human	2011
G1646-SLE-2011	KM821805.1	KM821806.1	Sierra Leone	Human	2011
G1529-SLE-2011	KM821801.1	KM821802.1	Sierra Leone	Human	2011
G1442-SLE-2011	KM821799.1	KM821780.1	Sierra Leone	Human	2011
G1200-LIB-2010	KM821797.1	KM821798.1	Sierra Leone	Human	2010
G1190-SLE-2010	KM821795.1	KM821796.1	Sierra Leone	Human	2010
G808-SLE-2010	KM821791.1	KM821792.1	Sierra Leone	Human	2010
G771-SLE-2010	KM821788.1	KM821789.1	Sierra Leone	Human	2010
G733-SLE-2010	KM821786.1	KM821787.1	Sierra Leone	Human	2010
G693-SLE-2009	KM821784.1	KM821785.1	Sierra Leone	Human	2009
G692-SLE-2009	KM821782.1	KM821783.1	Sierra Leone	Human	2009
G676-SLE-2009	KM821780.1	KM821781.1	Sierra Leone	Human	2009
G636-SLE-2009	KM821778.1	KM821779.1	Sierra Leone	Human	2009
G610-SLE-2009	KM821776.1	KM821777.1	Sierra Leone	Human	2009
G503-SLE-2009	KM821774.1	KM821775.1	Sierra Leone	Human	2009
G502-SLE-2009	KM821772.1	KM821773.1	Sierra Leone	Human	2009
Nig08-A19	GU481073.1	GU481074.1	Jos, Nigeria	Human	2008
Nig08-A18	GU481071.1	GU481072.1	Jos, Nigeria	Human	2008
Nig08-04	GU481069.1	GU481070.1	Abakaliki, Nigeria	Human	2008
Nig08-A47	GU481079.1	GU481080.1	Irrua, Nigeria	Human	2008
Nig08-A41	GU481077.1	GU481078.1	Irrua, Nigeria	Human	2008
Nig08-A37	GU481075.1	GU481076.1	Irrua, Nigeria	Human	2008
Bamba-R114	KF478761.1	KF478766.1	Bamba, Mali	*M. natalensis*	2012
Komina-R16	KF478760.1	KF478767.1	Komina, Mali	*M. natalensis*	2012
Soromba-R30	KF478763.1	KF478769.1	Soromba, Mali	*M. natalensis*	2012
Soromba-R	KF478762.1	KF478765.1	Soromba, Mali	*M. natalensis*	2009
NL	AY179172.1	AY179173.1	Sierra Leone	Human (Lethal)	2000
AV	AY179171.1	AY179172.1	Ghana/Ivory Coast	Human (Lethal)	2000
Z148	AY628204.1	AY628205.1	Zorzor, Liberia	Human (Non-lethal)	1981
Macenta	AY628200.1	AY628201.1	Liberia	Human (Lethal)	1984
BA366	GU979513.1	GU979514.1	Guinea	*M. natalensis*	2003
Josiah	HQ688674.1	HQ688672.1	Sierra Leone	Human (Lethal)	1976
Pinneo-NIG-1969	KM822127.1	KM822128.1	Lassa, Nigeria	Human (Non-lethal)	1969

### Phylogenetic analysis

Trees for LP, NP, and GPC were generated in MrBayes (Huelsenbeck and Ronquist, 2001; Ronquist and Huelsenbeck, 2003). The software utilizes a Bayesian Markov chain Monte Carlo (MCMC) algorithm to infer phylogenetic relationships. Parameters were set to utilize the invariant gamma rate of substitution model. Four chains (one hot chain and three cold chains) were utilized, and data was sampled every 100 steps. Each analysis was run for 10,000,000 steps with burn-ins set to 250,000 steps. Data was analyzed using Tracer version 1.6 (http://tree.bio.ed.ac.uk/software/tracer/) to confirm sufficient data sampling for each data set.

BEAST trees were generated for LP, NP, and GPC using BEAST (Drummond and Rambaut, 2007; Drummond et al., 2012). BEAST employs a Bayesian MCMC approach to infer demographic histories, evolutionary rates, and dates of divergence from serially (dated) sampled sequence data. Statistical uncertainty in the data is reflected in the 95% highest posterior density (HPD) values. Analyses were performed using the Bayesian Skyline Plot (BSP) model of population growth, which does not use a pre-specified demographic model (Drummond et al., 2005). The uncorrelated lognormal (UCLN) relaxed clock model, which allows rate variation among lineages in the phylogeny to be estimated (Drummond et al., 2006) was used. The MCMC chain was 100 million samples long, thinned to include every 5000th state in the final sample. The program Tracer version 1.6 was used to confirm stationarity. The software TreeAnnotator version 1.7.1 (http://beast.bio.ed.ac.uk/software/TreeAnnotator) was used to summarize the data output from BEAST. The maximum clade credibility (MCC) tree was estimated using mean node heights after discarding the initial 10% of generations.

## Results

### Bayesian analysis indicates the presence of a fifth LASV lineage

To begin, we first sought to determine whether there was evidence of a fifth lineage among LASV isolates using full-length NP sequences. The analysis that originally described the four classical lineages was based on partial-length NP sequences, but was later confirmed using full-length sequences from LP, NP, and GPC. While all four lineages were easily delineated from one another using full-length sequences, the relationship of each lineage to one another varied between genes (Bowen et al., 2000; Ehichioya et al., 2011). Therefore, in order to completely characterize the relationships of isolates within Mali and the Ivory Coast to the other classical isolates, we aligned the full-length open reading frames for GPC, NP, and LP and conducted a phylogenetic analysis utilizing the Bayesian MCMC approach (MrBayes v3.2.5) for each gene.

**Figure 1:**
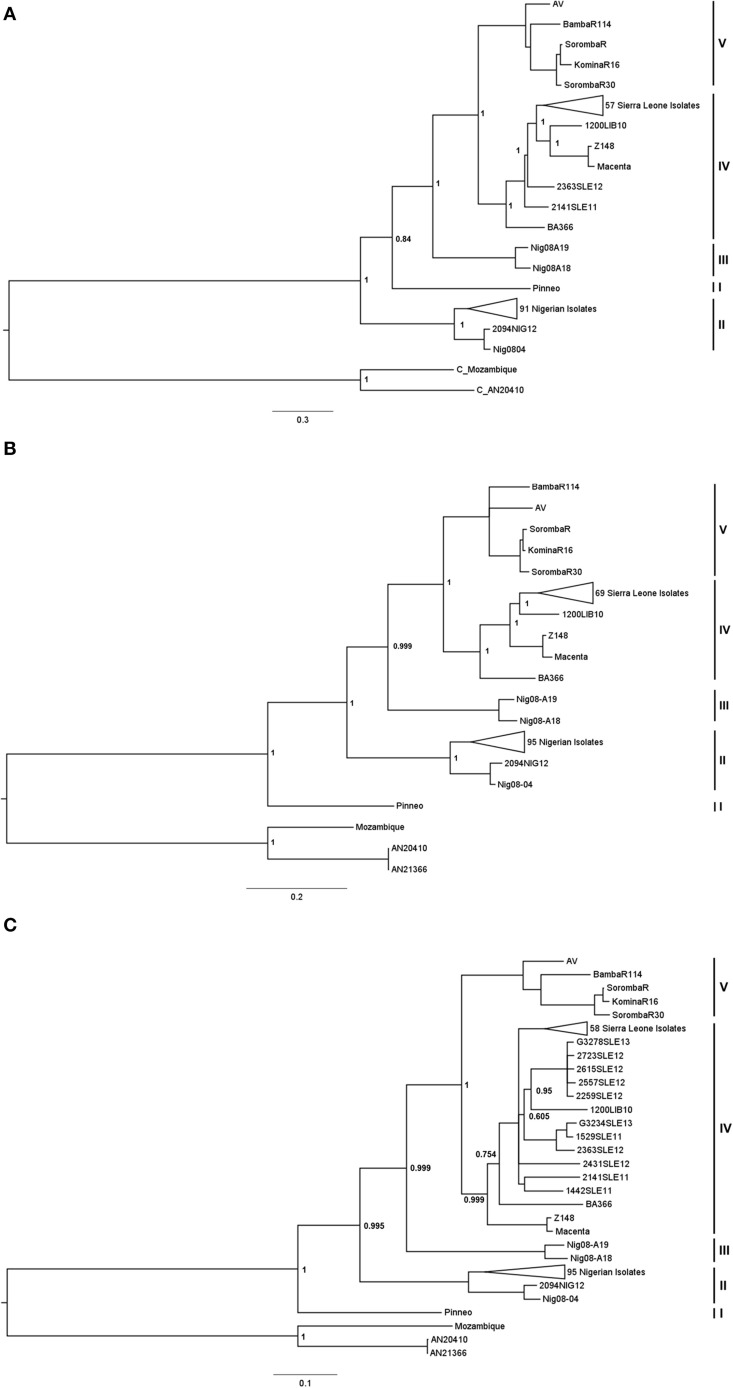
**Bayesian analysis of complete LP, NP, and GPC nucleotide sequences**. Complete open reading frames were aligned using MUSCLE, and analyzed using the Bayesian MCMC approach. The resulting trees for LP **(A)**, NP **(B)**, and GPC **(C)** were visualized using FigTree v1.4.2 and rooted using two representative Mopeia virus isolates. To better visualize distinct lineages, several Sierra Leonian, and Nigerian isolates were collapsed. These sections of the trees are provided in Figures S1–S6. Posterior probabilities are represented as node labels for the main clades, with 1 being 100% probability. The isolates are grouped by their respective lineages, as represented by the bars to the right of the trees. The scale represents nucleotide changes per site per year.

Analysis of both the NP and GPC genes produced phylogenetic trees (Figure 1) that resemble the traditional grouping of the four lineages, with lineage I (Pinneo) creating the most basal lineage. The hierarchy continues with lineage II, lineage III (Nig08-A18, Nigo8-A19), and lineage IV. In both trees, the isolates from Mali and the Ivory Coast delineate from lineage IV distinctly with strong bootstrap support. However, analysis of full-length LP nucleotide sequences (Figure 1) places lineage II as the most basal lineage, followed by lineage I, lineage III, and lineage IV.

Within lineage IV, the Liberian isolates (Z148, Macenta, 1200LIB10) cluster together in only the LP analysis. The isolate 1200LIB10 however does share a recent common ancestor with Z148 and Macenta in the NP analysis despite being most closely related to the Sierra Leone isolates. Interestingly, the 1200LIB10 isolate clusters within the Sierra Leone isolate clade in the GPC analysis. The Guinea rodent isolate BA366 is basal to the Liberia and Sierra Leone isolates in the NP analysis, but shares a more recent common ancestor with the Sierra Leone strains with respect to Z148 and Macenta in the GPC analysis.

Five strains of LASV fell into a different grouping, designated Lineage V, these strains included AV (Ivory Coast/Ghana), BambaR114 (Mali), KominaR16 (Mali) SorombaR (Mali), and SonombaR30 (Mali). These form a single well defined lineage with high posterior probability support. All the isolates from this lineage were isolated from Mali and the Ivory Coast suggesting that this lineage is geographically restricted, maybe due to either geographical barriers or distribution of a distinct haplotype of the rodent host *M. natalensis*.

### The fifth LASV lineage emerged during the colonial period

**Figure 2:**
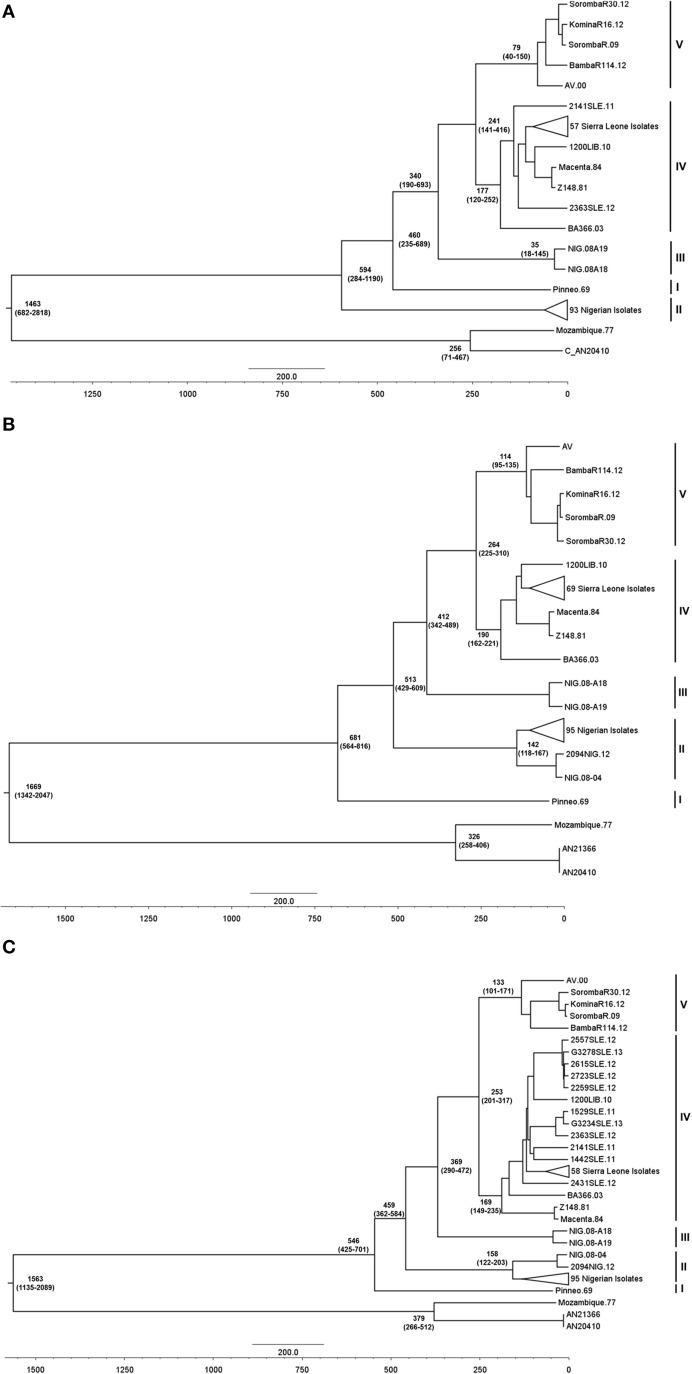
**BEAST analysis of complete LP, NP, and GPC nucleotide sequences**. Complete open reading frames were aligned using MUSCLE, and analyzed using the Bayesian MCMC approach. The resulting trees for LP **(A)**, NP **(B)**, and GPC **(C)** were visualized using FigTree v1.4.2 and rooted using two representative Mopeia virus isolates. To better visualize distinct lineages, several Sierra Leonian and Nigerian isolates were collapsed, and these sections of the trees are provided in Figures S7–S12. The node ages, in years, are included on the major nodes, with the 95% confidence ranges displayed in parentheses below the median node ages. The isolates are grouped by their lineages, as represented by the bars to the right of the trees. The reverse axis represents the age, in years, from the most recent isolate.

In order to determine when the lineage emerged, we performed a BEAST analysis using the trees obtained from MrBayes. By providing the year of isolation for each isolate, we were able to approximate the emergence of lineages IV and V from their nearest common ancestor. Analysis of all three complete genes estimates the emergence of lineage V to have occurred roughly 250 years ago (Figure 2), which coincides with movement throughout the region during the colonial period of West Africa. While both S segment genes estimate the most recent common ancestor between lineages IV and V to have existed between 200 and 300 years ago, the estimated range is much larger in the LP analysis (141–416 years ago). The most recent common ancestor of the lineage V group was approximately 114 years ago, with a range of 225–30.

Movement of the virus from Nigeria to the Mano river region is predicted to have occurred between roughly between 300 – 500 years ago with respect to the S segment genes (Figure 2). However, the range for the LP gene is much larger (190–693 years ago). Western movement of LASV from Nigeria likely occurred during the pre-colonial period of West Africa between the years 1500 and 1700 AD, although the virus appears to have been circulating in Nigeria prior to 1300 AD. Additionally, the most recent common ancestor of LASV and Mopeia virus (MOPV) is estimated to have existed between 0 and 700 AD based on S segment gene analysis (Figure 2). The origin of LASV in the Nigerian region is most likely, but the sampling of LASV is still heavily biased in favor of certain regions and therefore increased sampling is required to fully determine the origins and movements of this virus.

## Discussion

This study represents the first phylogenetic analysis of LASV that includes every available isolate from the traditional four lineages, as well as every available isolate from Mali and the Ivory Coast. While the lineage hierarchy for both the GPC and NP supports the results from the original analysis (Bowen et al., 2000), lineage I is not the most basal lineage in the LP analysis. However, this is not strongly supported by the posterior probability suggesting that until more strains are isolated it will be difficult to resolve with the LP gene. It is possible for two closely related arenaviruses to reassort (Lukashevich, 1992). It is possible that a reassortment event occurred between ancestral lineage I and lineage II strains, but as previous studies have not detected any reassortment events among LASV strains (Vieth et al., 2004; Emonet et al., 2006) this seems unlikely. However, the lineage I isolate was not available at the time. Our results indicate that lineage I remains the most basal lineage based on full-length GPC analysis. However, these findings do not support the previous findings by Ehichioya et al., which places lineage II as the most basal lineage for GPC. Evidence of recombination between arenavirus species has been described within the New World arenaviruses (Fulhorst et al., 1999; Weaver et al., 2000), which could explain different groupings between two genes in the same segment. However, no evidence of recombination was detected in their analysis (Ehichioya et al., 2011). It is possible that the different alignment method utilized prior to our analysis contributed to the different outcome.

The discrepancy between the GPC gene and the NP and LP genes in the topology of lineage IV is likely due to the additional number of strains belonging to lineage IV in the GPC gene tree. As full genome sequences of these strains become available we would expect these observed differences to be resolved.

Analysis of all three full-length genes supports the emergence of a fifth LASV lineage, which appears to have diverged from a common ancestor with lineage IV around 250 years ago. Conflict situations and the resulting human movement have been described to perturb the virus relationship with its peridomestic natural host, *M. natalensis*. Movement of *M. natalensis* over large distances, such as transportation by ship or even through movement of refugees during conflicts, can lead to foci of transmission among the local *M. natalensis* population (Lalis et al., 2012). Emergence of the fifth lineage may have therefore occurred due to human movement during the colonial period.

**Figure 3:**
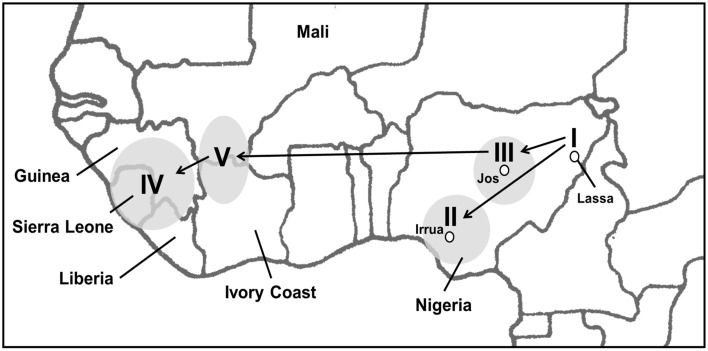
**Map of LASV movement across West Africa**. Based on the phylogenetic data, LASV has gradually spread west, beginning in Eastern Nigeria. Ehichioya et al. has previously illustrated the movement of LASV within Nigeria (Ehichioya et al., 2011). The isolates of lineage V and the BA366 Liberian isolate share a more direct common ancestor with lineage III than the Sierra Leone Isolates share with lineage III isolates. This suggests that the virus was likely present in Mali, the Ivory Coast, and Liberia prior to establishing itself in Sierra Leone. The areas from which the isolates were collected are shaded in gray.

When comparing the AV strain to the Sierra Leone strains, the AV strain is more closely related to the Liberian BA366 strain and the Nigerian lineage III strains. Based on this relationship between the Nigerian isolates and the isolates of lineages IV and V, it appears likely that the virus spread gradually west, establishing focal points of transmission in the Ivory Coast and Mali prior to its arrival in Liberia, Guinea, and Sierra Leone. Although the prediction varies between the three genes, this migration likely occurred during the pre-colonial and colonial periods, possibly arriving in Mali and the Ivory Coast between 300 and 450 years ago and Guinea or Liberia around 250 years ago. This is supported by the substitution rate estimates previously calculated, suggesting that the spread of LASV across West Africa occurred between 300 and 800 years ago (Ehichioya et al., 2011). Andersen et al. recently performed a similar phylogenetic analysis using a large number of complete L segment sequences. Their conclusions were almost identical to our own, indicating a gradual movement of LASV across West Africa (Andersen et al., 2015). In both analyses, the virus is predicted to have arrived in Mali and the Ivory Coast a full century prior to its arrival in Sierra Leone (Figure 3).

In conclusion, this study reports that a fifth LASV lineage exists within Mali and the Ivory Coast, sharing a sister relationship with the isolates of lineage IV. Despite the apparent presence of the virus within Mali over the last 200 years, it is peculiar that LF cases have only begun to surface within the last decade. The recent emergence of reported cases may be due to the lack of surveillance in the region, particularly in villages with limited access to healthcare. Nevertheless, the presence of a genetically distinct LASV lineage within this region will likely serve to increase the genetic variability in an already diverse virus species. These findings highlight the importance of considering genetic diversity among LASV isolates when developing and testing treatments and vaccine candidates.

### Conflict of interest statement

The authors declare that the research was conducted in the absence of any commercial or financial relationships that could be construed as a potential conflict of interest.
