# Supplementary figures and images for: Lassa virus isolates from Mali and the Ivory Coast represent an emerging fifth lineage
Source: Front Microbiol. 2015 Oct 1;6:1037. doi: 10.3389/fmicb.2015.01037 (PMC4589679; doi:10.3389/fmicb.2015.01037)

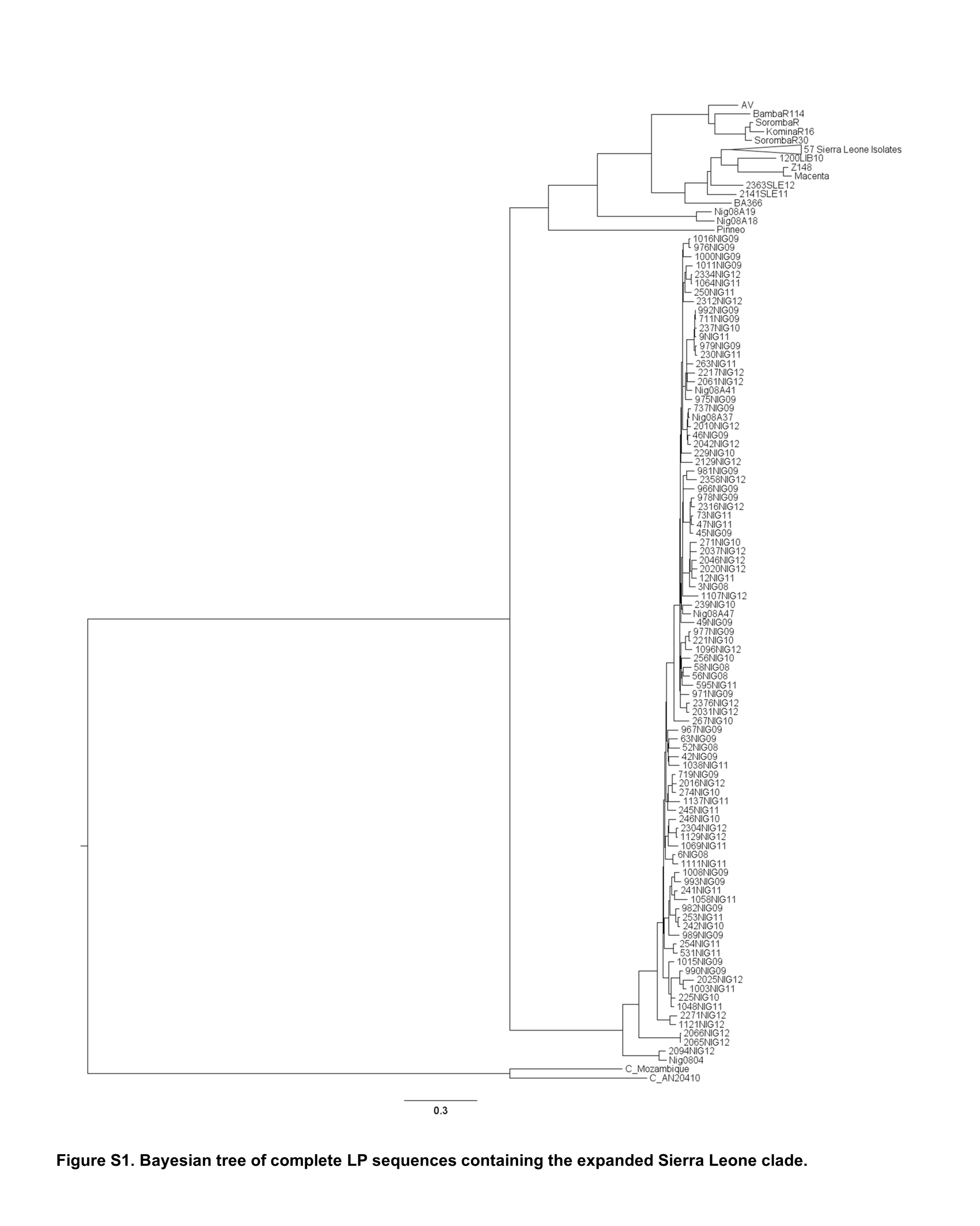

Supplement: Supplementary file 1 [file Image1.TIF]

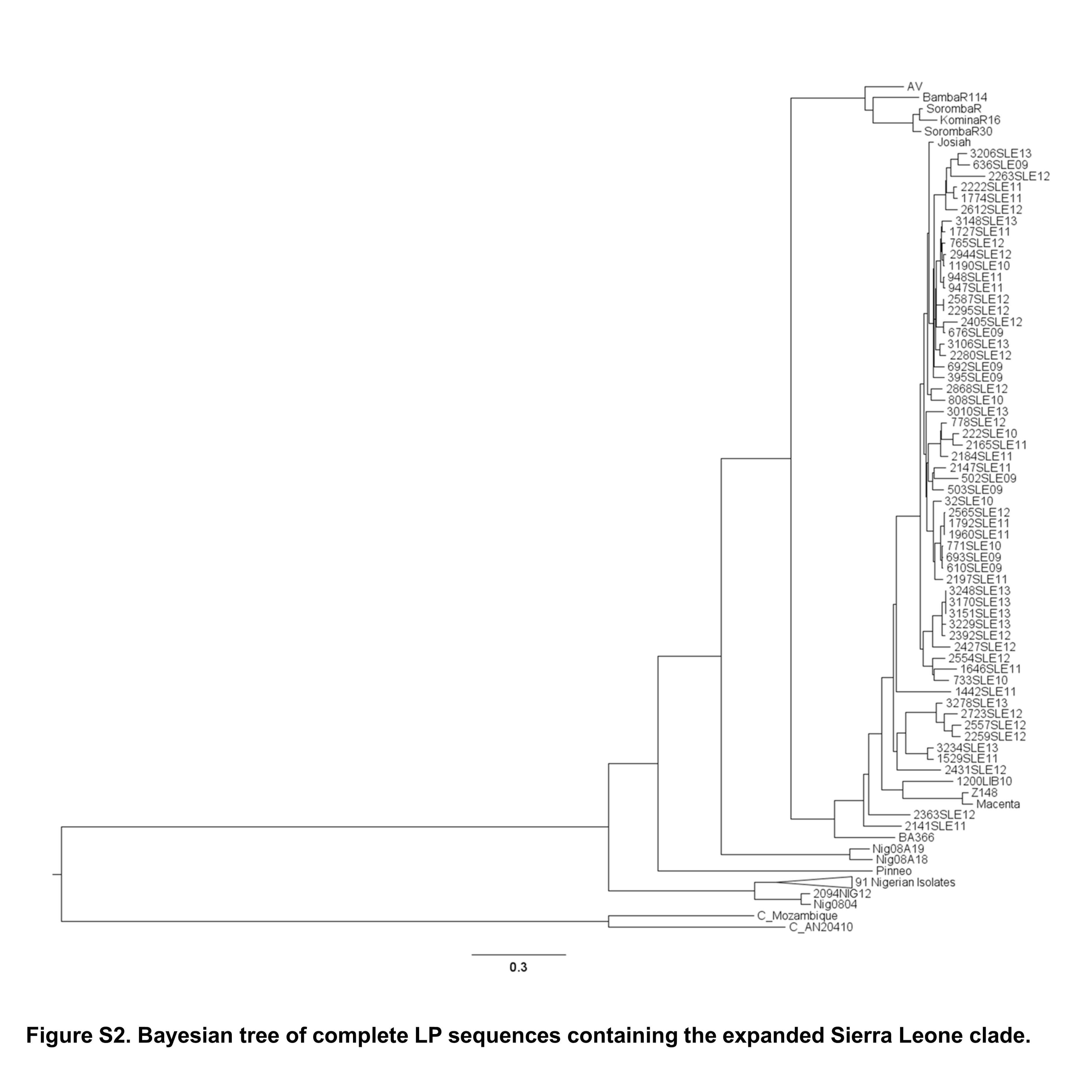

Supplement: Supplementary file 2 [file Image2.TIF]

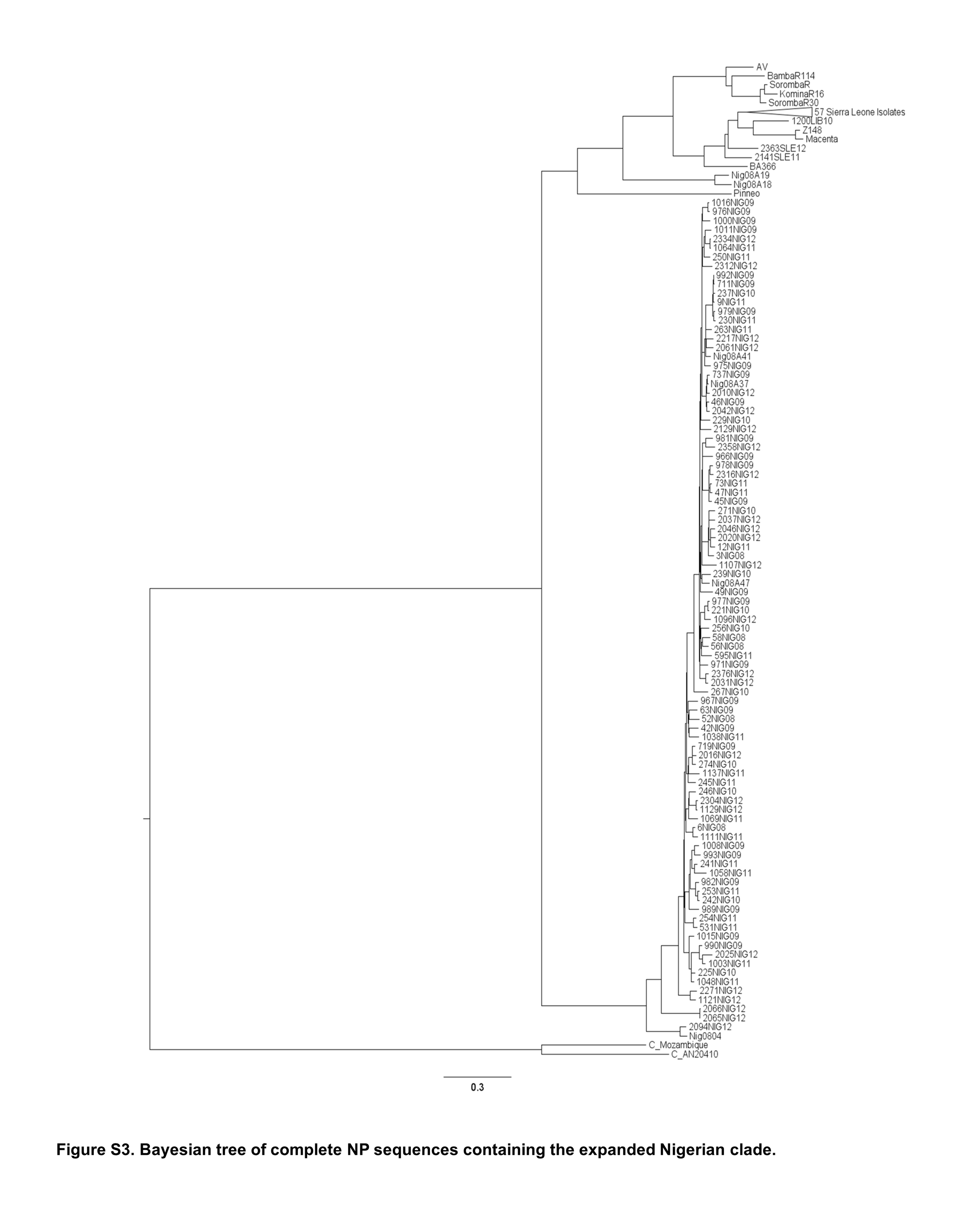

Supplement: Supplementary file 3 [file Image3.TIF]

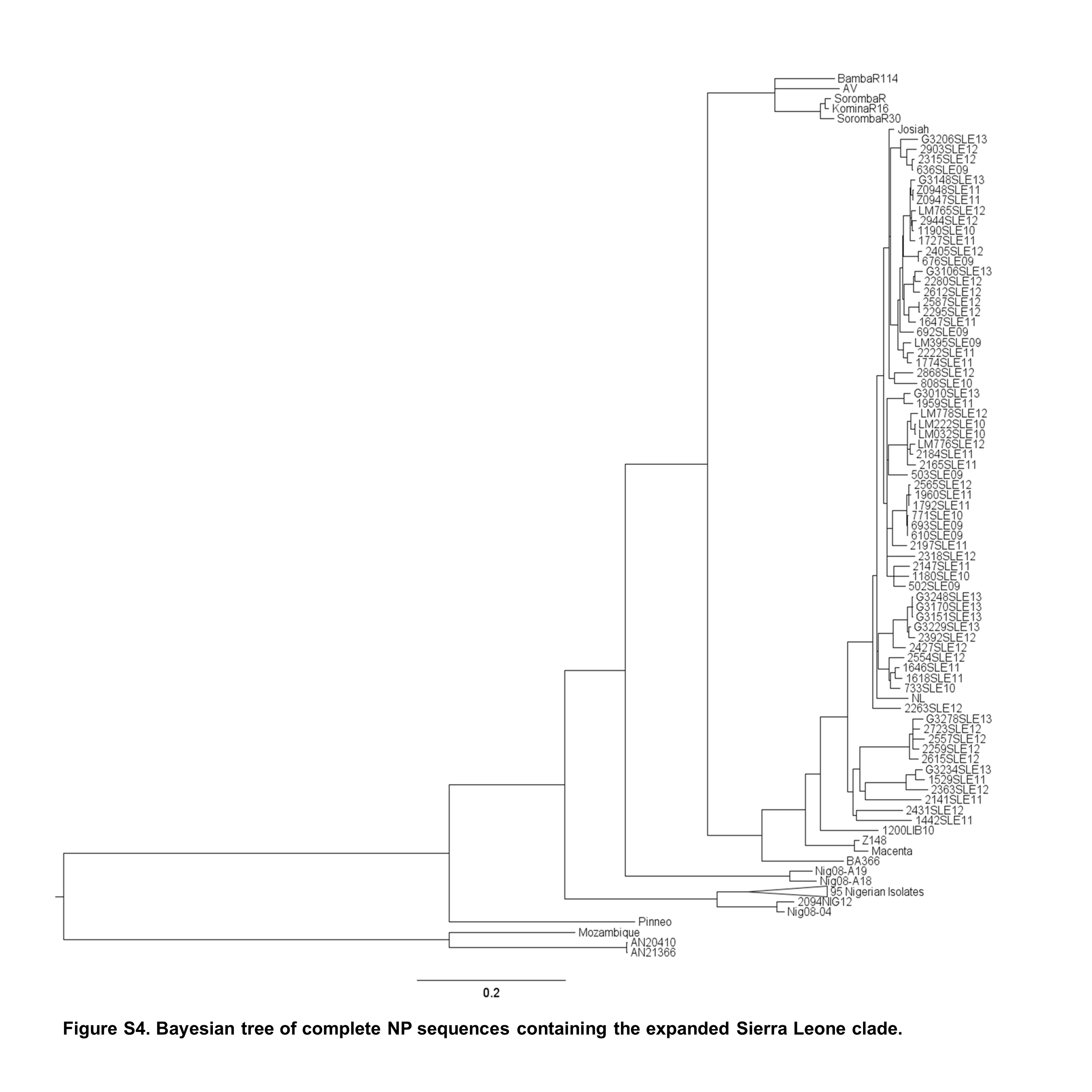

Supplement: Supplementary file 4 [file Image4.TIF]

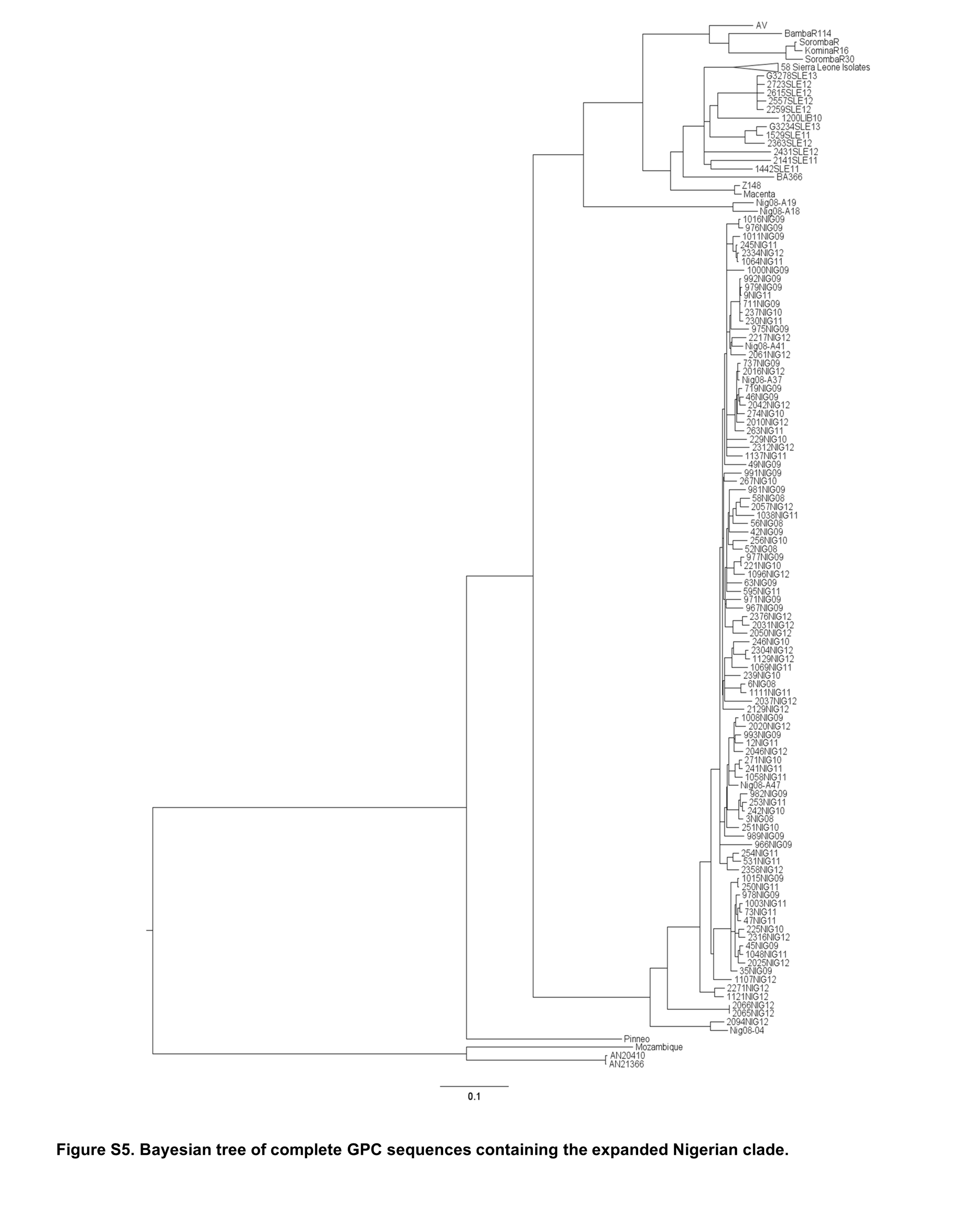

Supplement: Supplementary file 5 [file Image5.TIF]

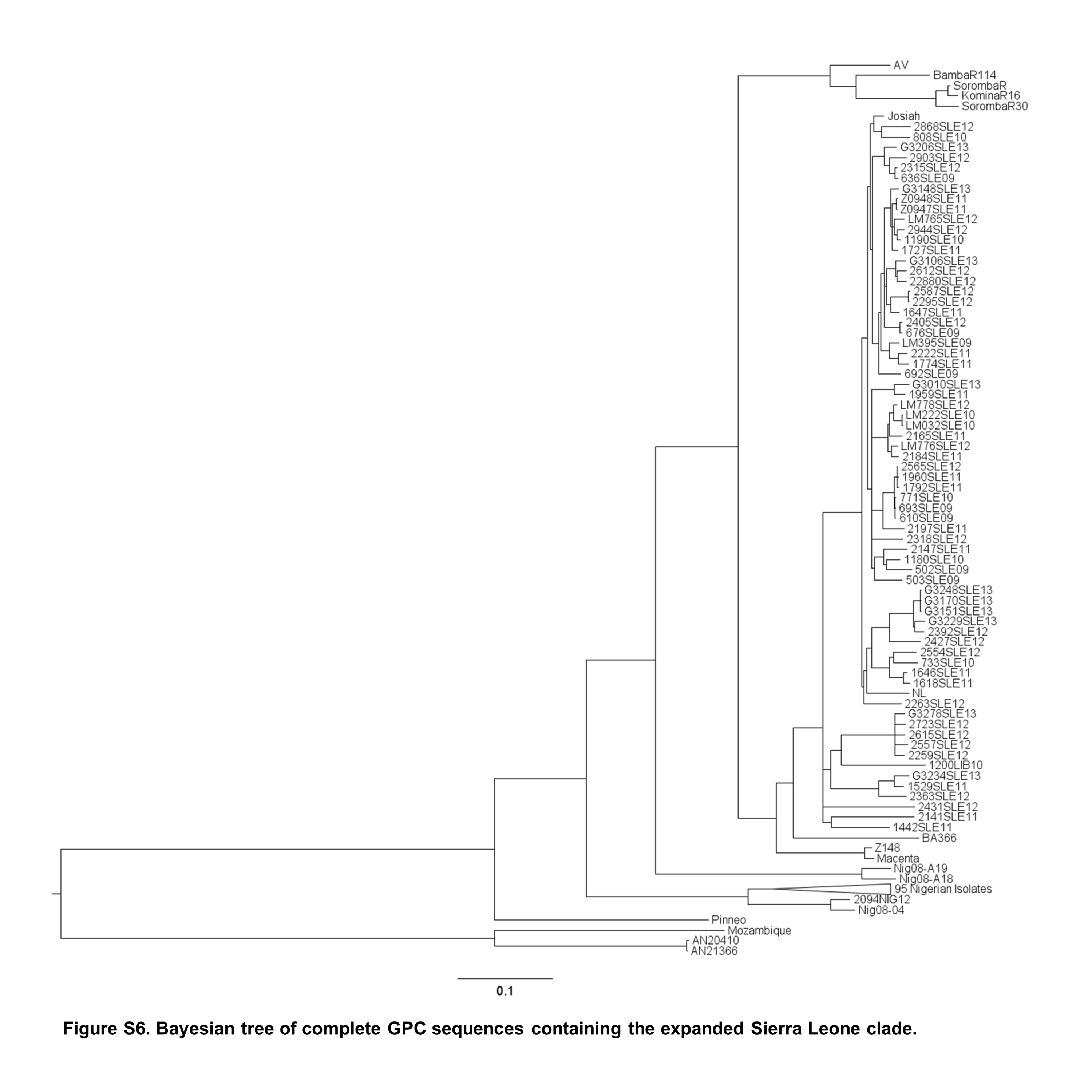

Supplement: Supplementary file 6 [file Image6.TIF]

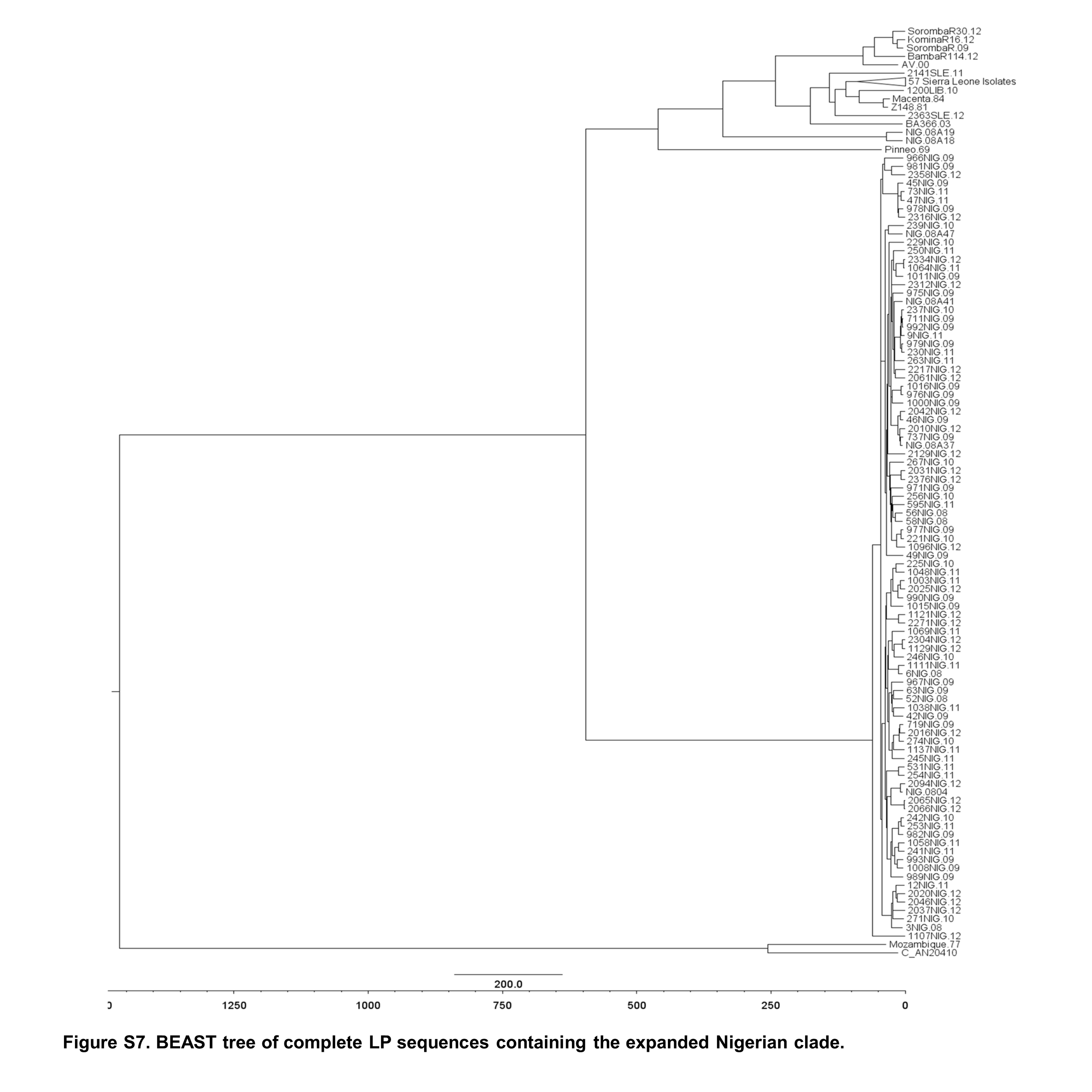

Supplement: Supplementary file 7 [file Image7.TIF]

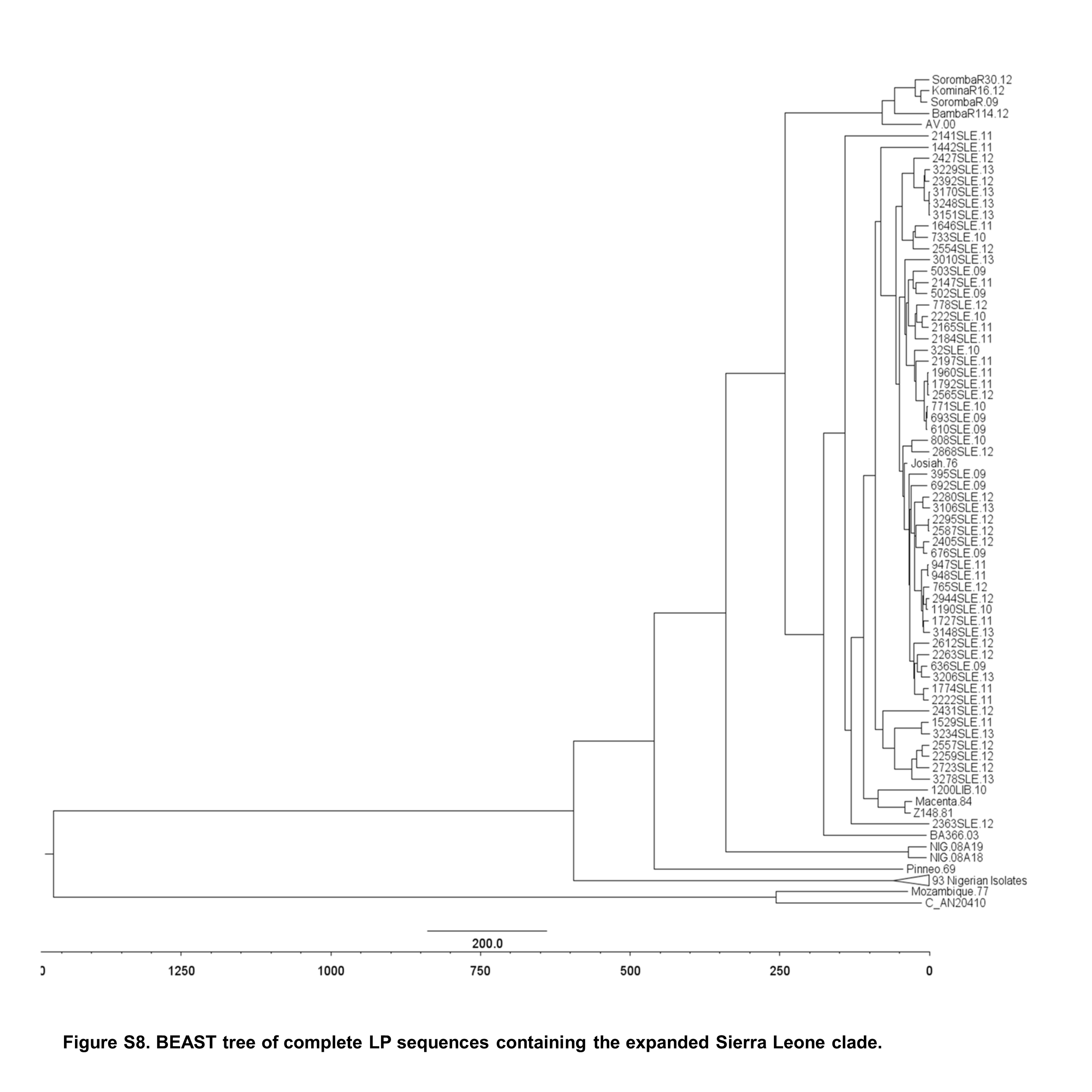

Supplement: Supplementary file 8 [file Image8.TIF]

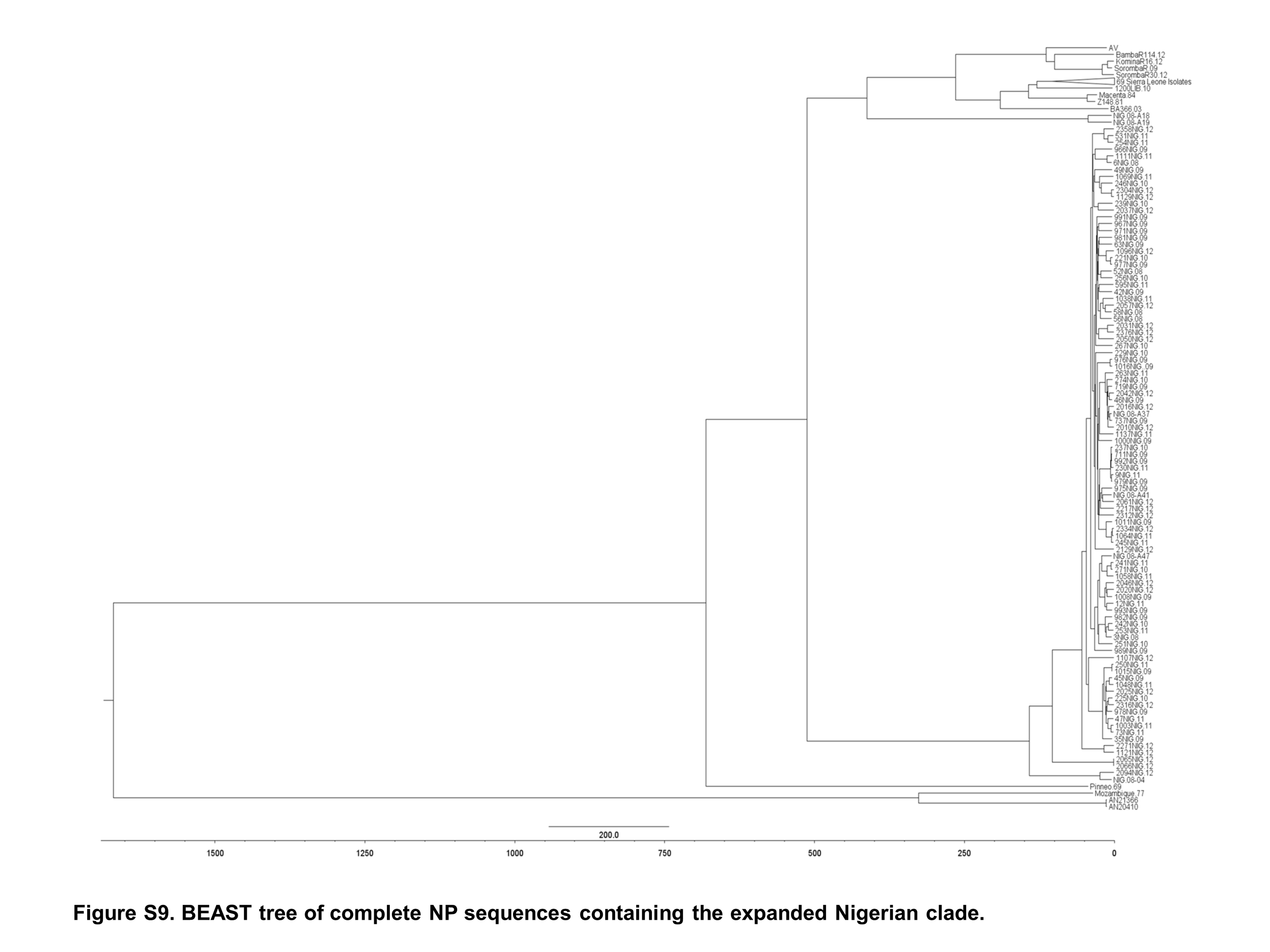

Supplement: Supplementary file 9 [file Image9.TIF]

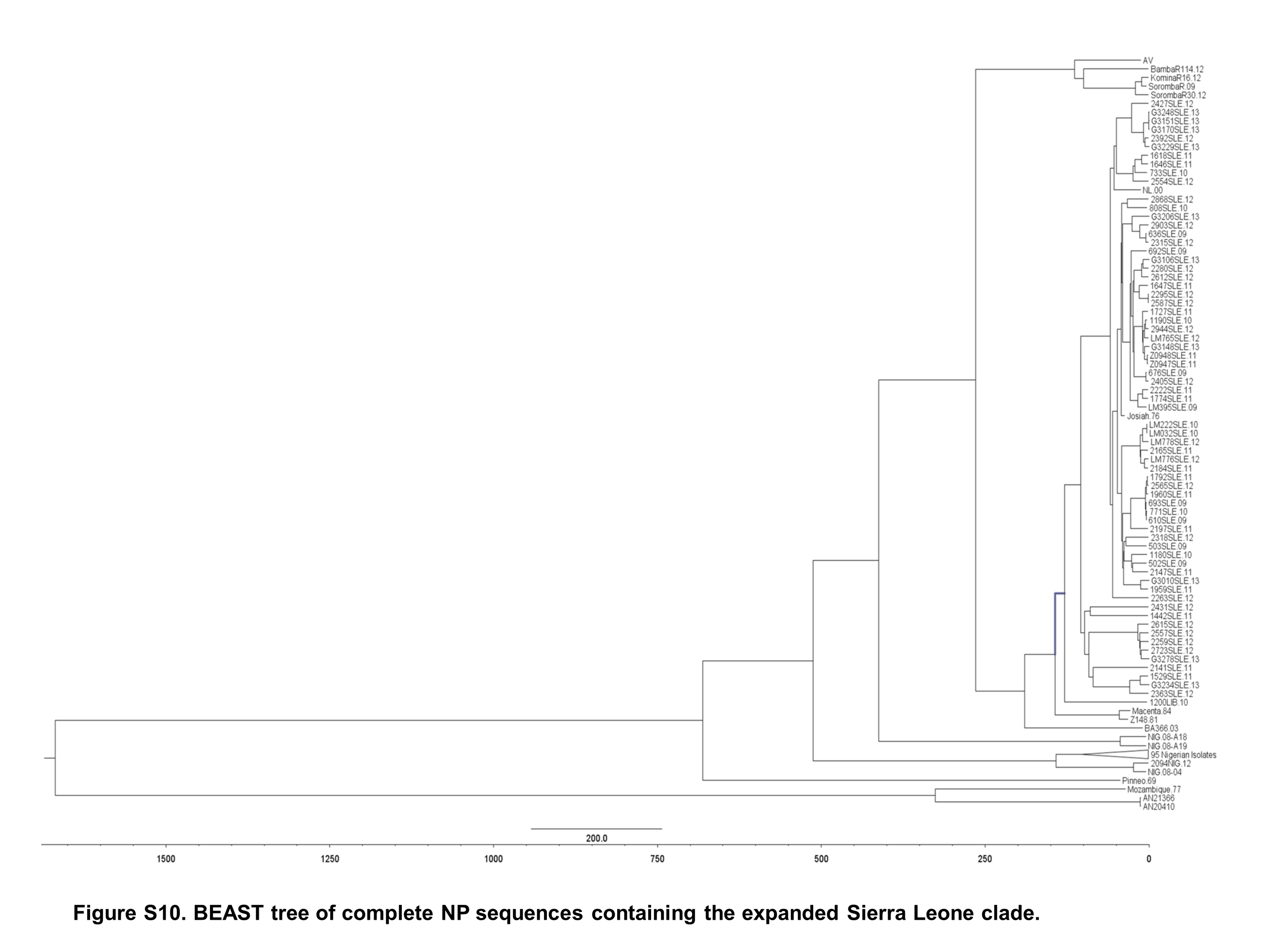

Supplement: Supplementary file 10 [file Image10.TIF]

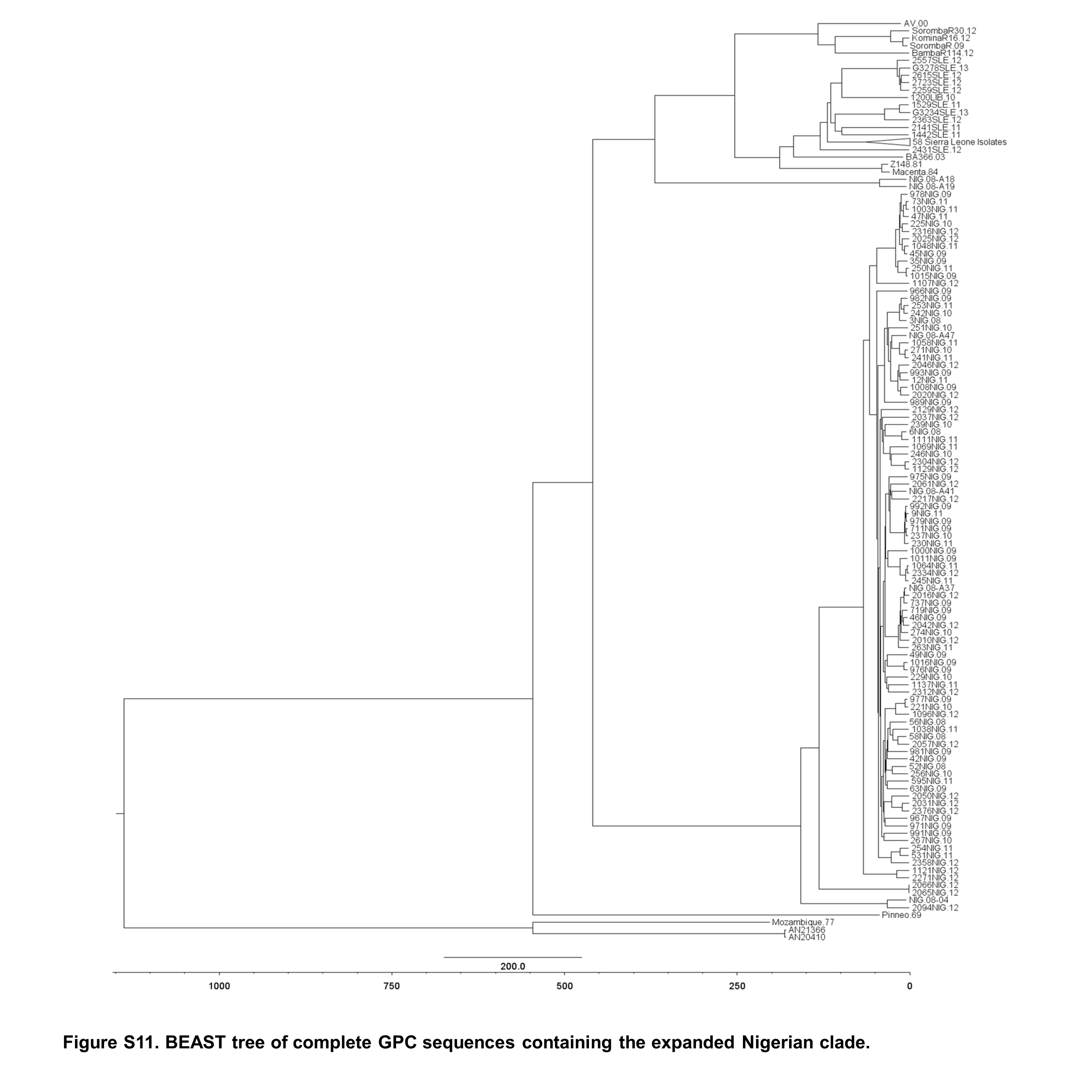

Supplement: Supplementary file 11 [file Image11.TIF]

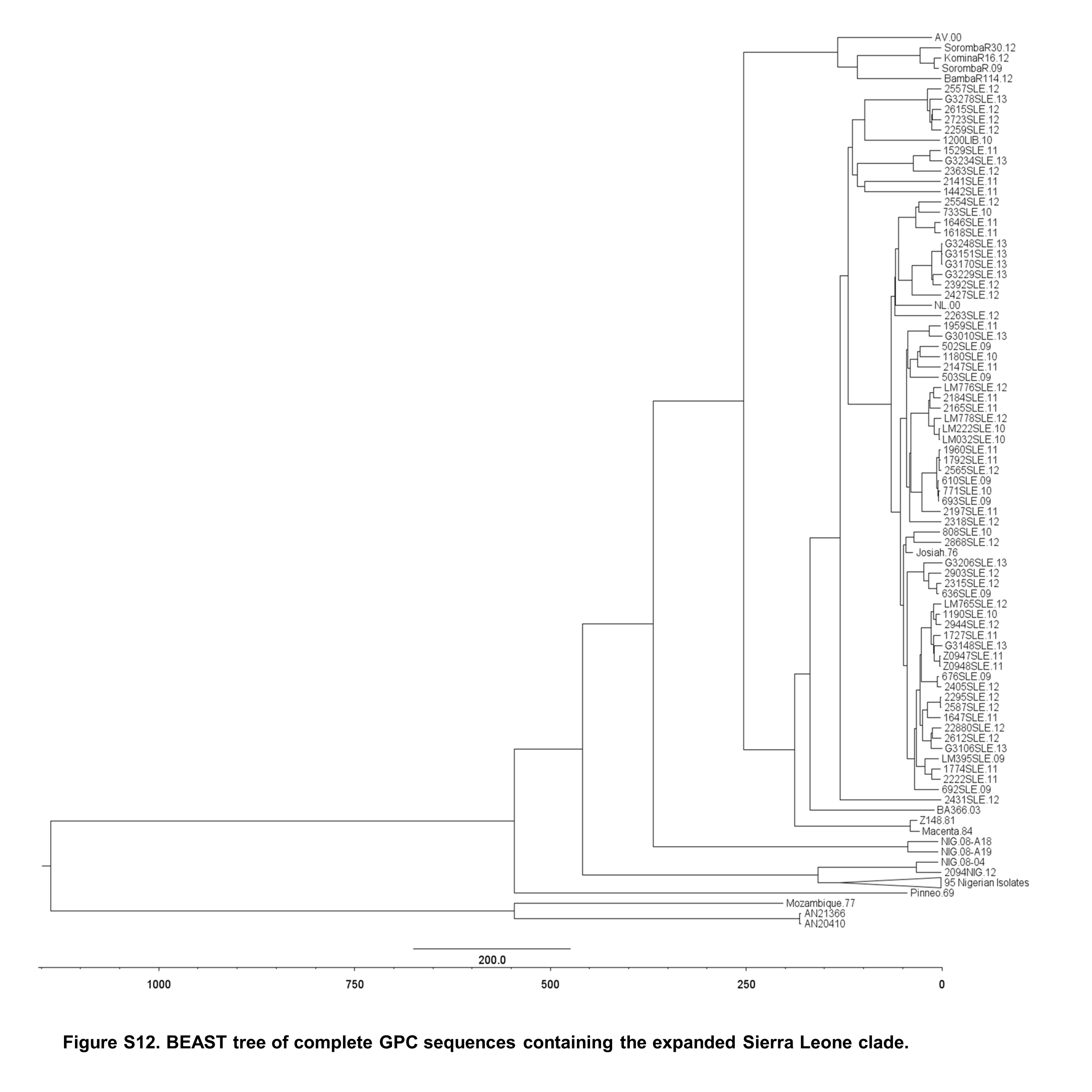

Supplement: Supplementary file 12 [file Image12.TIF]
